# Modulation of Autophagy: A Novel “Rejuvenation” Strategy for the Aging Liver

**DOI:** 10.1155/2021/6611126

**Published:** 2021-02-10

**Authors:** Fengming Xu, Hans-Michael Tautenhahn, Olaf Dirsch, Uta Dahmen

**Affiliations:** ^1^Department of General, Visceral and Vascular Surgery, Jena University Hospital, Jena 07747, Germany; ^2^Institute of Pathology, Klinikum Chemnitz gGmbH, Chemnitz 09111, Germany

## Abstract

Aging is a natural life process which leads to a gradual decline of essential physiological processes. For the liver, it leads to alterations in histomorphology (steatosis and fibrosis) and function (protein synthesis and energy generation) and affects central hepatocellular processes (autophagy, mitochondrial respiration, and hepatocyte proliferation). These alterations do not only impair the metabolic capacity of the liver but also represent important factors in the pathogenesis of malignant liver disease. Autophagy is a recycling process for eukaryotic cells to degrade dysfunctional intracellular components and to reuse the basic substances. It plays a crucial role in maintaining cell homeostasis and in resisting environmental stress. Emerging evidence shows that modulating autophagy seems to be effective in improving the age-related alterations of the liver. However, autophagy is a double-edged sword for the aged liver. Upregulating autophagy alleviates hepatic steatosis and ROS-induced cellular stress and promotes hepatocyte proliferation but may aggravate hepatic fibrosis. Therefore, a well-balanced autophagy modulation strategy might be suitable to alleviate age-related liver dysfunction. *Conclusion*. Modulation of autophagy is a promising strategy for “rejuvenation” of the aged liver. Detailed knowledge regarding the most devastating processes in the individual patient is needed to effectively counteract aging of the liver without causing obvious harm.

## 1. Introduction

Life expectancy of the population increased substantially. This is due to the development of medical technology and general improvement of sanitary conditions, resulting in an increase of the aging population. In 2019, there were about 703 million (9%) people aged 65 and above in the world. This figure is expected to almost double to 1.5 billion (16%) by 2050 [1].

Age is one of the important risk factors for malignant liver disease. Aging causes changes in hepatic morphology, structure, and function with hepatic steatosis, fibrosis, and impaired liver regeneration being the most prominent features [2–5].

The liver is the pivotal metabolic organ, which is involved in central metabolic activities such as lipid metabolism, gluconeogenesis, and protein synthesis [6, 7]. The age-related changes do not only impair the function of the liver but also represent a potential risk for the occurrence of malignant liver diseases [2]. Therefore, clinicians face the problem of how to eliminate or mitigate aging-related detrimental changes in the liver.

Autophagy is a crucial mechanism for eukaryocytes to recycle intracellular constituents. During the process of autophagy, misfolded proteins or defective organelles are degraded to basal components via the lysosomal pathway for later reuse [8]. Autophagy contributes to liver homeostasis through its role in ATP synthesis and organelle quality control [9]. However, the level of hepatic autophagy gradually decreases with age [10, 11]. Aging affects autophagy mainly via inhibition of adenosine monophosphate-activated protein kinase (AMPK) activation, hypermethylation of autophagy-related genes, and accumulation of lipofuscin. Lipofuscin is an intracellular brown-yellow pigment granule, which accumulates within the lysosomal compartment during cellular senescence [12–19]. If damaged cellular components or excess reactive oxygen species (ROS) accumulate in cells, cellular homeostasis is disrupted and cellular senescence is further accelerated [20].

In the last ten years, the role of autophagy in liver diseases has attracted more and more attention. Accumulating evidence shows that promoting autophagy effectively mitigates hepatic steatosis, restores impaired liver regeneration, reduces mitochondrial dysfunction, and alleviates ROS-induced cellular injury. However, it may exacerbate the progress of hepatic fibrosis. In this review, we describe the role of autophagy in age-associated liver changes and suggest how to modulate autophagy to rejuvenate the aging liver.

## 2. Autophagy

Autophagy is an intracellular degradation process. The autophagy-related genes control the process in which eukaryocytes digest their damaged or superfluous components such as misfolded proteins, damaged organelles, and pathogens via the lysosomal pathway [8, 21]. It conveys a prosurvival effect allowing cells to maintain energy homeostasis and accommodate cellular stressors such as excess ROS, anoxia, and nutrient starvation [21, 22]. In contrast, excessive autophagy may lead to cell death [23].

**Figure 1:**
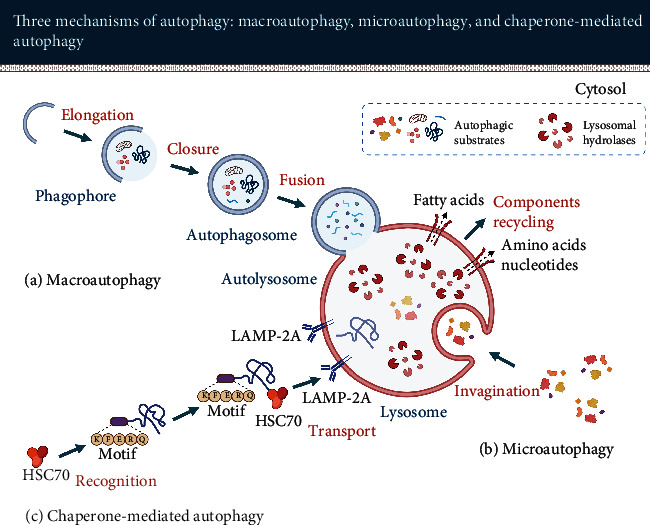
Three key types of autophagy in eukaryocytes: macroautophagy, microautophagy, and chaperone-mediated autophagy. They all ultimately transport autophagic substrates to the lysosomes for degradation through different pathways prior to releasing the resulting building blocks such as amino acids, fatty acids, and nucleotides back into the cytosol for cellular reuse.

Autophagy can be classified into macroautophagy, microautophagy, and chaperone-mediated autophagy (CMA). The classification is based on the delivery route of autophagy substrates involving different morphological features. All three types of autophagy ultimately deliver substrates to lysosomes for degradation and reutilization (see Figure 1) [24, 25].

### 2.1. Different Types of Autophagy

#### 2.1.1. Macroautophagy

**Figure 2:**
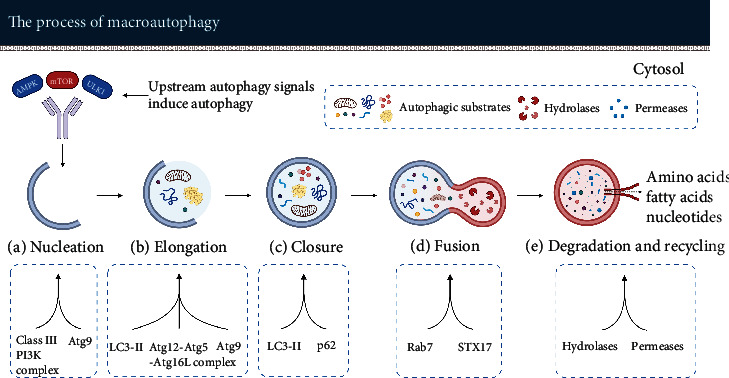
Macroautophagy degrades aggregated intracellular proteins or damaged organelles thereby providing the raw material for the production of new intracellular organelles (component-recycling system). (a) The class III PI3K complex induces the initiation of phagophore formation. Atg9 promotes membrane transport from the donor organelle to the phagophore. (b) With the joint action of LC3, Atg12-Atg5-Atg16L complex, and Atg9, the phagophore elongates gradually. (c) LC3 promotes closure of the membranes. p62 interacts with autophagic substrates and delivers the substrates to autophagosomes under the control of LC3. (d) Rab7 and STX17 facilitate the fusion of autophagosome and lysosome. (e) Autophagic substrates are degraded by the action of lysosomal hydrolases into small molecules that are subsequently released by membrane permeases for use in the construction of new organelles.

*The first step* of macroautophagy is the nucleation of phagophore (see Figure 2). Activation of autophagy signaling molecules such as AMPK, mammalian target of rapamycin (mTOR), or Unc-51 like autophagy activating kinase 1 (ULK1) initiates the process.

The nucleus of the phagophore is derived from a subdomain of the endoplasmic reticulum (ER) called omegasome. Omegasomes are rich in phosphatidylinositol-3-phosphate (PI3P, a crucial lipid messenger for autophagy initiation) [26, 27]. When nucleation is complete, the phagophore enters a rapid growth phase. The most critical step in this phase is membrane acquisition. Phagophores get in contact with other organelles such as plasma membranes [28–30], mitochondria [31], and Golgi complex [32, 33] which may serve as potential membrane sources [34, 35]. Membranes are transported from the donor organelle to the phagophore via Atg9, a crucial transmembrane protein [36].

**Figure 3:**
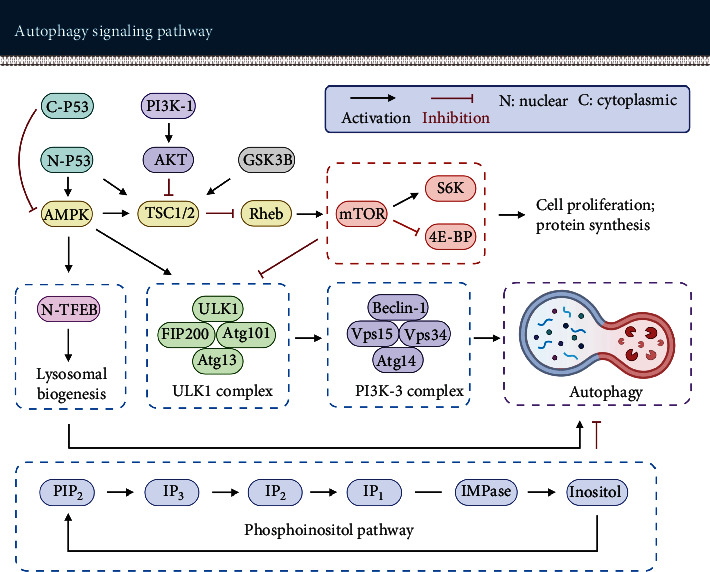
Autophagy (encircled in purple) is modulated by multiple signaling pathways; they can be divided into mTOR-dependent (encircled in red) and mTOR-independent autophagy signaling pathways (encircled in blue). mTOR is a central regulatory molecule of autophagy and cell growth. Inducing autophagy by inhibiting mTOR activity impedes protein synthesis and cell proliferation.

One of the key regulators that promote the nucleation of phagophore is the ULK1 complex. This complex is composed of ULK1, FAK family kinase interacting protein of 200 kDa (FIP200), autophagy-related protein 13 (Atg13), and Atg101 (see Figure 3) [37]. ULK1, a serine/threonine kinase, has several downstream phosphorylation targets to promote the formation of phagophores. FIP200 is supposed to act as a “scaffolding molecule” in the ULK1 complex. Atg13 acts as an adaptor in the complex to facilitate the interaction between ULK1 and FIP200, and Atg13 boosts the activity of ULK1. Atg101 plays an essential role in the stability and phosphorylation of Atg13 and ULK1 [38, 39]. Moreover, Atg101 promotes the recruitment of downstream autophagic proteins [40].

The ULK1 complex phosphorylates the components of the class III PI3K (PI3K-3) complex. The PI3K-3 complex is composed of Beclin-1, vacuolar protein sorting 34 (Vps34), Vps15, and Atg14 (see Figure 3). Beclin-1 is a core constituent of the PI3K-3 complex. The phosphorylation of Beclin-1 via ULK1 is considered to be required for activation of the Atg14-bound Vps34 [41]. Vps34 produces PI3P in phagophores and stabilizes the ULK1 complex. Vps15 is essential for activation and maintaining the function of Vps34 [40, 42]. Atg14 targets the PI3K-3 complex to the phagophore assembly site and promotes the extension of phagophores [40].

*The second step* of macroautophagy is autophagosome formation consisting of elongation and closure of the phagophore. The phagophores continuously elongate and capture autophagic substrates in the cytosol. Ultimately, the phagophores form sealed double-membrane autophagosomes.

Microtubule-associated protein light chain 3 (LC3) is a vital protein in this process. Cytosolic LC3 (LC3-I) is covalently bound to phosphatidylethanolamine (PE) to form lipidated LC3-II under the mediation of the Atg12-Atg5-Atg16L complex. The Atg12-Atg5 conjugate enhances the activity of Atg3 to promote the transfer of LC3 from Atg3 to PE. Atg16L specifies the site of the LC3-lipidation reaction. LC3-II locates at the inner and outer membrane and is crucial for the expansion and closure of the isolation membrane [43–45]. Sequestosome-1 (SQSTM1, also known as p62) is an autophagy receptor that recruits autophagic substrates. It interacts with LC3 on the isolation membrane via the LC3 interaction region and targets it to the autophagosome [45–47].

*The third step* of macroautophagy is the fusion of autophagosomes with lysosomes. Autophagosomes usually fuse with lysosomes directly. However, they can also fuse with late endosomes to form intermediate autophagic vacuoles called amphisomes which then fuse with lysosomes to form autolysosomes [48, 49].

Ras-related protein in brain 7 (Rab7) is a small GTPase that is located in lysosomes and late endosomes [48]. It is one of the key enzymes of membrane trafficking. For autophagy, Rab7 promotes autophagosome clustering in the perinuclear area and the fusion of autophagosomes with lysosomes, but the detailed molecular mechanism is still unclear [50–54]. Syntaxin 17 (STX17) facilitates the fusion process as well. STX17 is a SNARE protein that is located in the outer membrane of completed autophagosomes. It interacts with synaptosomal-associated protein 29 (SNAP-29) and vesicle-associated membrane protein 8 (VAMP8) to form a STX17 complex. This complex facilitates the fusion of autophagosomes and lysosomes [48, 55].

*The final step* of macroautophagy is degradation and recycling of the enclosed autophagic substrates. The autophagic substrates such as sequestered organelles and aggregated proteins are degraded in autolysosomes via multiple lysosomal hydrolases. After degradation, the resulting monomers such as amino acids, fatty acids, and nucleotides are released to the cytosol through the action of lysosomal permeases for cell reutilization [56].

Depending on the specific autophagy substrate, macroautophagy can be further classified into lipophagy and mitophagy [57], described in detail below. There are also other forms such as pexophagy, nucleophagy, and ribophagy, which we are not explaining here.

The term “lipophagy” is used to describe the process of autophagic degradation for lipid droplets (LDs) [58]. Lipophagy was first observed in the fasting liver and is an important process in lipid metabolism. It contributes to lipid turnover not only in liver cells but also in various other animal cells [59, 60]. Under normal physiological conditions, lipophagy is regulated by the nutritional status of cells via AMPK-mTOR pathways. During periods of starvation, lipophagy is activated, allowing cells to utilize their fat reserves [58].

Similar to the above, mitophagy is the process of selective degradation for damaged or redundant mitochondria via macroautophagy [61, 62]. Mitophagy plays a crucial role in mitochondrial quality control and regeneration [63, 64]. Impaired mitophagy disrupts mitochondrial function, leading to the progressive accumulation of defective mitochondria and eventual cell damage [62]. The Parkin-PINK1 pathway is the critical pathway regulating mitophagy. In general, activated PINK1 facilitates Parkin to bind with depolarized mitochondria to induce mitophagy [65]. We will describe the mechanism of mitophagy mediated by this pathway in detail in a subsequent section (Section 7.2).

#### 2.1.2. Microautophagy

Unlike in macroautophagy, microautophagy does not involve autophagosomes as a vehicle for transporting autophagy substrates. In microautophagy, intracellular substances are directly engulfed by the lysosome membrane via invagination and then degraded within the lysosomal lumen [66, 67]. The major function of microautophagy is to maintain membrane homeostasis and organelle size and promote cell survival under nitrogen restriction [68].

#### 2.1.3. Chaperone-Mediated Autophagy

Chaperone-mediated autophagy regulates the degradation of a selective population of cytosolic proteins containing a specific KFERQ peptide sequence [69]. It is estimated that about 30% of cytosolic proteins contain this sequence motif [70].

Firstly, the molecular chaperone heat-shock cognate protein of 70 kDa (HSC70) recognizes and binds the substrate protein. In a second step, the substrate proteins are transported into the lysosome under the mediation of lysosome-associated membrane protein type 2A (LAMP-2A) for degradation [71]. The selectivity of chaperone-mediated autophagy results in degradation of specific motif proteins only without interfering with other types of proteins. Chaperone-mediated autophagy mainly facilitates protein homeostasis and promotes cellular adaptation to stress [72].

Here, we mainly focus on macroautophagy which is the most relevant form of autophagy within the hepatic aging process.

### 2.2. Autophagy Participates in a Variety of Physiological Metabolic Activities in the Liver

The role of autophagy in liver physiology was discovered during the past ten years. The main findings can be summarized as follows:

Under homeostatic condition: firstly, hepatic autophagy degrades lipid droplets into free fatty acids (FFAs) which are oxidized in mitochondria to promote ATP synthesis [6, 73]. It facilitates the energy homeostasis of hepatocytes. Secondly, hepatic autophagy promotes the removal of damaged organelles. The accumulation of abnormal organelles leads to hepatocyte swelling and hepatotoxicity [66, 74, 75]. Thirdly, autophagy breaks down misfolded proteins into amino acids which are used in the synthesis of new proteins [76]. Hepatic autophagy may also convert amino acids to glucose via gluconeogenesis, which is an essential process for maintaining blood glucose concentration [7].

Under stress conditions: the liver maintains a basal level of autophagy which is substantially enhanced in response to cellular metabolic stress. For example, starvation-induced autophagy occurs primarily in the liver [6, 7]. A study on perfused rat livers showed that under basal-nutrient conditions, the rate of protein degradation is about 1.5% of total liver protein per hour, while under starvation, this rate could be increased to about 4.5% [77].

Upon impairment of autophagy process: a number of reports have shown that liver-specific autophagy deficiency leads to significant hepatomegaly and liver injury in animals [74, 75, 78]. In 2-month-old Atg5-deficient mice, the liver to body weight ratio (LBWR) was about 2-fold that of control mice, and the serum alanine aminotransferase (ALT) level was about 8-fold higher [78]. These changes reflect the important role of autophagy in the liver, which may be related to the accumulation of abnormal organelles caused by autophagy deficiency [74, 75].

Upon aging: hepatic autophagy activity gradually decreases with age [10, 11, 79, 80], which may set the stage for the occurrence of age-related liver diseases.

### 2.3. Autophagic Activity Declines with Age

Aging, the process of becoming older, leads to spontaneous and inevitable changes in the structure and function of organism over time. This is mainly manifested in the degeneration of biological structures, the decline in physiological functions, and the reduction of stress adaptation [81, 82].

Aging leads to a decline of autophagy in a variety of tissues such as the liver, brain, and ovary [10, 83–85]. For instance, in aged mouse liver, the LC3 protein expression, the number of hepatocytes with autophagic vacuoles, and the total number of autophagic vacuoles in hepatocytes are substantially reduced [86].

#### 2.3.1. Aging Impairs the Activation Capacity of AMPK

AMPK, the major energy-sensing kinase, activates various catabolic processes. AMPK activation can effectively induce the initiation of the autophagic process. Activated AMPK triggers autophagy to facilitate energy generation in mitochondria and downregulates energy-demanding processes such as cell division and protein synthesis to ensure cellular energy homeostasis [87].

However, aging results in a significant decrease in the activation capacity of AMPK. Reznick et al. [16] observed in the skeletal muscle of old rats that activation of AMPK induced by acute (5′-aminoimidazole-4-carboxamide-1-*β*-D-ribofuranoside, exercise) or chronic (*β*-guanidinopropionic acid) stimulation was significantly reduced compared with young rats.

The age-related impairment of AMPK activation impedes autophagosome formation, affects cellular homeostasis, and further promotes the aging process via weakening its inhibitory effect on mTOR [17, 18, 88].

#### 2.3.2. Age-Related Lipofuscin Accumulation Impairs the Degradation Efficiency of Lysosome

Lipofuscin is a brown-yellow and autofluorescent pigment mainly composed of oxidated protein and lipid residues [89]. The formation of lipofuscin is primarily due to iron-catalyzed oxidation of protein and lipid macromolecules [90, 91]. Lipofuscin typically accumulates in the lysosomes of postmitotic cells during senescence.

Lysosomes are acidic organelles that contain multiple hydrolytic enzymes. When lysosomes loaded with lipofuscin accumulate in senescent cells, most of the lysosomal enzymes are drawn from the Golgi apparatus to the lipofuscin-loaded lysosomes. However, lysosomal enzymes degrade proteins but are unable to degrade lipofuscin. As a result, the delivery of enzymes to lipofuscin-loaded lysosomes is ineffective for recycling the aggregated proteins. This imbalanced distribution reduces the availability of lysosomal enzymes in healthy lysosomes, leading to a marked decrease in the lysosomal degradation process [13–15].

Furthermore, the decreased turnover of dysfunctional mitochondria (impaired mitophagy) due to lipofuscin accumulation leads to a substantial increase in the generation of reactive oxygen species (ROS). In turn, the increased oxidative stress impairs autophagy via further impairment in lysosomal function [92–96].

#### 2.3.3. Aging Facilitates Hypermethylation of Autophagic Genes

As mentioned before, Atg5 and LC3 are pivotal genes governing the autophagic process [45, 97]. So far, it was shown in two different compartments, macrophages and ovaries, but not yet in the liver, that age-related hypermethylation of Atg 5 and LC3 did lead to a downregulation of autophagy.

Khalil et al. [19] observed that mRNA expression of Atg5 and LC3B was significantly reduced in bone marrow-derived macrophages of aged mice. The promoter regions of Atg5 and LC3B were highly methylated compared to those in young mice. Preventing methylation via methyltransferase inhibitor, (2)-epigallocatechin-3-gallate (EGCG), or DNA methyltransferase 2 (DNMT2) siRNA restored the expression of Atg5 and LC3B in the macrophages of aged mice. Li et al. [83] observed also age-related hypermethylation of autophagic genes, albeit in mouse ovaries: the mRNA and protein expression of Atg5 and LC3B were significantly decreased in the ovaries of aged rats. The promoter regions of Atg5 and LC3B were highly methylated compared to those in young rats. The authors pointed out that the observed upregulation of DNA methyltransferase 3A/3B in the ovaries of aged rats may lead to methylation of Atg5 and LC3B, which in return may ultimately decrease autophagy activity.

These results suggest that aging may blunt autophagy activity by promoting the hypermethylation of autophagic genes.

### 2.4. Main Pathways to Modulate Autophagy

We first give an explanation of the key molecules regulating autophagy: mTOR and its related complexes mTORC1 and mTORC2. In the second step, we describe the autophagy regulatory pathways.

mTOR is the major regulator of autophagy. It is a serine-threonine kinase, which is involved in the regulation of multiple cellular activities such as autophagy, cell growth, proliferation, and metabolism [98]. The level of mTOR expression is negatively correlated with the activity of autophagy, e.g., inhibition of mTOR induces autophagy remarkably. mTOR can interact with several binding proteins to form two different protein complexes that are referred to as mTOR complex 1 (mTORC1) and mTOR complex 2 (mTORC2). The activity of mTOR is regulated by multiple upstream factors such as AMPK, AKT, and TSC1/2. Phosphorylation of AMPK and TSC1/2 inhibits the activity of mTORC1, while AKT phosphorylation promotes the activity of mTORC1 [99–101].

mTORC1, a rapamycin-sensitive protein complex, is involved in regulating autophagy, cell growth, protein synthesis, and ribosome biosynthesis [87, 98]. mTORC1 is regulated by AMPK, which is actually one of the most important upstream modulators of mTORC1. mTORC1 senses the cellular energy status through AMPK. In addition, mTORC1 can sense the level of other cellular nutrients as well such as amino acids, growth factors, and oxygen. mTORC1 has three important downstream effectors: p70-S6 kinase (S6K), 4E-binding protein (4E-BP), and ULK1. S6K1 and 4E-BP are closely related to the regulation of protein synthesis and cell growth [87, 98, 102], while ULK1 is an important regulator of autophagosome formation (see Figure 3). In nutrient-rich conditions, mTORC1 is activated and promotes cell growth and proliferation by phosphorylating S6K and 4E-BP. In contrast, activated mTORC1 phosphorylates and inactivates of ULK1 to suppress autophagy [103–106].

mTORC2, a rapamycin-insensitive protein complex, mainly regulates cell survival and modulates the actin cytoskeleton to organize the cell shape [87, 98, 107]. According to Saxton and Sabatini, the critical role of mTORC2 is to phosphorylate and activate AKT which facilitates cellular survival and growth [98]. This view was further confirmed by Kazyken et al. [108]. Kazyken et al. observed that the activation of AMPK phosphorylated and activated mTORC2 in hepatocytes. In his experiments, AMPK-mediated activation of mTORC2 was not induced by AMPK-mediated inhibition of mTORC1, but that AMPK directly phosphorylated mTORC2. The activation of AMPK by starvation stimulated mTORC2 and its substrate AKT to facilitate cell survival. By contrast, inactivation of AMPK, mTORC2, and AKT aggravated cell apoptosis during starvation.

Now, we describe four critical autophagy modulating pathways.

#### 2.4.1. PI3K-AKT-mTOR

Phosphoinositide 3-kinase (PI3K) is an intracellular phosphatidylinositol kinase. PI3K is involved in a series of cellular events such as autophagy, apoptosis, and proliferation. PI3K activation can effectively activate AKT. AKT is a serine/threonine protein kinase, it plays an important role in cell growth, proliferation, and survival [109]. mTOR acts as a downstream molecule of the PI3K-AKT pathway.

Activation of PI3K by phosphorylation results in the production of a second messenger-phosphatidylinositol-3,4,5-triphosphate (PIP3), which binds to PDK1 (phosphoinositide-dependent kinase-1) and AKT. PDK1 phosphorylates and activates AKT. There are three ways for activated AKT to regulate mTOR. First, AKT phosphorylates mTOR directly, thereby activating mTOR and inhibiting autophagy. Second, AKT can phosphorylate and inactivate proline-rich AKT substrate of 40 kilodaltons (PRAS40), a downstream target of AKT that inhibits the activity of mTORC1, as well, thereby activating mTORC1. Third, AKT enriches the Ras homolog enriched in the brain (Rheb) via phosphorylating tuberous sclerosis complex 1/2 (TSC1/2). Activated Rheb activates mTOR to inhibit autophagy (see Figure 3) [101, 110–114].

#### 2.4.2. AMPK-mTOR-ULK1

As mentioned above, AMPK is considered to be a central cellular energy sensor. It is activated in response to energy stress [87].

AMPK regulates mammalian autophagy in two ways. First, AMPK phosphorylation of TSC2 leads to the inactivation of Rheb, which in turn leads to the inactivation of mTOR. mTOR inactivation restores the activity of ULK1 which is a critical initiator of autophagy. Second, AMPK can phosphorylate ULK1 directly, which in turn facilitates the formation of autophagosomes [12, 99, 104, 115–120].

#### 2.4.3. p53-AMPK-mTOR

p53 is a tumor suppression protein. It is mainly considered as a DNA sequence-specific transcription factor, which is involved in activating proapoptosis, cell-cycle arrest, and proautophagy genes [121, 122].

Emerging evidence suggests that p53 may bidirectionally regulate autophagy based on its subcellular localization. The active p53 tetramer in the nucleus binds to the promoter regions of multiple pro-autophagy-related genes such as AMPK, TSC2, and damage-regulated autophagy modulator (DRAM) to transactivate the expression of proautophagy genes, thereby inducing autophagy [121, 123]. For example, nuclear p53 can trigger autophagy in a DRAM (a lysosomal protein that induces macroautophagy)-dependent way [124]. Furthermore, nuclear p53 can inhibit mTOR via the phosphorylation and activation of AMPK to induce autophagy [125], whereas cytoplasmic p53 inhibits autophagy [123, 126].

Furthermore, p53 plays an important role in cell senescence and proliferation. Activated p53 triggers the expression of its downstream prosenescence molecules such as p21 and E2F Transcription Factor 7 (E2F7). p21 is a cyclin-dependent kinase (CDK) inhibitor that leads to p53-dependent cell-cycle arrest and induces cell senescence. E2F7 is a transcriptional repressor of E2F target genes and is substantially upregulated during cellular senescence. E2F7 inhibits the expression of mitogenic genes and cooperates with retinoblastoma protein (RB) to promote cell cycle arrest [123, 127–130].

#### 2.4.4. Phosphoinositol Pathway

Inositol or inositol 1,4,5-trisphosphate (IP_3_) elimination can induce autophagy as well [131, 132]. The activation of autophagy may be related to the role of Ca^2+^ in energy metabolism.

The ER stores most of the intracellular Ca^2+^. After IP_3_ binds to the membrane IP_3_ receptor on the ER surface, Ca^2+^ can be released from the ER. This process is thought to be a requirement for maintaining the energy state of mitochondria since providing Ca^2+^ to mitochondria promotes the production of nicotinamide adenine dinucleotide (NADH) and energy. Conversely, inhibition of IP_3_ or IP_3_ receptors will result in a decrease in energy production. The reduced energy level stimulates AMPK and triggers autophagy through an mTOR-independent mechanism to maintain cellular energy balance [12, 133, 134].

## 3. Age-Related Common Alterations in the Liver

The liver is the largest solid organ of the human body, and it is mainly composed of 4 types of cells: hepatocytes, hepatic stellate cells (HSCs), Kupffer cells (KCs), and liver sinusoidal endothelial cells (LSECs).

Hepatocytes are the major parenchymal and functional cells of the liver, accounting for about 70% of the total liver cells. They perform multiple functions such as metabolic (lipid, carbohydrate, and protein), detoxifying (xenobiotics), and secretory (bile) functions to ensure metabolic homeostasis.

The remaining 30% of hepatic cells are primarily HSCs, LSECs, and KCs [135, 136]. HSCs are mainly involved in the storage of vitamin A in lipid droplets (LDs) and regulation of extracellular matrix and may affect sinusoidal blood flow via their contractile properties [4, 137]. LSECs constitute a permeable barrier within the liver sinusoids. They can promote the exchange of substances between the blood flow in the sinusoids and the surrounding tissues [138, 139]. KCs are resident hepatic macrophages. They are considered to act as “pathogen-scavengers” that play a major role in the immune and inflammatory response of the liver [140].

### 3.1. The Influence of Aging on Liver Cells

With age, the number of hepatocytes gradually decreases, the genome of hepatocytes becomes unstable, and the number of polyploid hepatocytes increases. Moreover, lipofuscin accumulation and mitochondrial dysfunction also appear in senescent hepatocytes [4, 141].

In addition, the number of HSCs increases, and the number of activated stellate cells, staining positive for *α*-smooth muscle actin (*α*SMA, a stellate cell activation marker), increases as well [4]. In LSECs, aging leads to a decrease in the number and size of fenestrations, an increase in the deposition of basal collagen, and thickening of the endothelium [142] compromising the intercellular molecular exchange. Moreover, the implications of aging on macrophages include a decrease in phagocytosis and an increase in the secretion of cytokines that lead to an inflammatory phenotype [4].

### 3.2. The Influence of Aging on Liver Morphology and Structure

The molecular changes described above also lead to changes on the macroscopical level. Age-associated accumulation of lipofuscin in hepatocytes leads to a gradual change in the color of the liver from light brown to dark brown [143].

Age-associated decrease in the number and quality of hepatocytes seems to cause a gradual decrease in the size and perfusion of the liver [4, 143]. Wynne et al. [144] reported a reduction of more than 40% when comparing a young with an old liver (24 years versus 91 years).

Aging also affects hepatic morphology and liver regeneration as mentioned before. Steatosis and fibrosis progressively appear in the aged liver [2–5]. A number of authors (see Tables 1–4) are giving evidence that there is a close link between autophagy and age-related diseases of the liver. Autophagy plays an important role in these hepatic diseases such as nonalcoholic fatty liver disease (NAFLD) and hepatic cirrhosis but also in posthepatectomy liver failure due to inadequate liver regeneration.

## 4. Liver Steatosis

The liver is the major organ of lipid metabolism. Hepatic lipid metabolism is of central importance for the synthesis, storage, secretion, and catabolism of triglycerides and fatty acids [145]. Liver steatosis occurs upon disturbances of the hepatic lipid metabolism, e.g., increased lipid synthesis or decreased lipid degradation in the liver. Steatosis can induce progressive hepatic pathological alterations, including lobular inflammation, ballooning degeneration, and fibrosis [146–149].

### 4.1. Aging is Associated with Development of Hepatic Steatosis

The lipid metabolism capacity of the liver graduate declines with age [150]. Steatosis can be observed in mouse livers above the age of 12 months [151]. Steatosis and especially nonalcoholic fatty liver disease (NAFLD) are also often observed in the human elderly population. NAFLD is characterized as the presence of more than 5% of fat-laden hepatocytes in the absence of a competing cause of liver steatosis [147]. According to a 2012 Rotterdam study, the overall prevalence of NAFLD was 35.1% in the elderly population aged over 65 years old [152]. NAFLD may progress to nonalcoholic steatohepatitis (NASH), liver cirrhosis, and eventually liver cancer without effective intervention [153].

### 4.2. Hepatic Steatosis Also Impairs Autophagy

As mentioned before, the autophagy activity declines with age [10, 11, 80]. The age-related accumulation of hepatic lipids, described above, further impairs autophagic activity. This was nicely illustrated in the study of Inami et al. [154]. They observed in a mouse model of genetically induced obesity (ob/ob mouse) that the p62 expression level was significantly increased in the steatotic liver compared to the control group. Furthermore, the rate of degradation for long-lived proteins, the activity of cathepsin B/L (lysosomal proteases), and the ratio of lysotracker red-stained autophagosomes were significantly lower in hepatocytes from ob/ob mice compared to control mice. These results suggest that hepatic steatosis impaired autophagy by impeding autophagosome acidification and expression of proteolytic enzymes.

Moreover, autophagy is involved in the regulation of cellular energy and nutrient metabolism. Conversely, energy and nutrient levels modulate autophagy as well. For example, during a period of starvation, lipophagy is activated to provide the needed FFAs for ATP synthesis. In contrast, adequate nutrition inhibits lipophagy since cells do not need FFAs as energy sources [58]. Overnutrition and obesity can activate mTOR by inactivating AMPK, which may blunt the ULK1 kinase complex and in turn inhibit autophagy [155, 156].

### 4.3. Impaired Lipophagy Contributes to Accumulation of Lipid Droplets in Hepatocytes

The process of converting lipid droplets into FFAs, called lipolysis, includes two different types: neutral lipolysis and acid lipolysis (also referred to as lipophagy). Neutral lipolysis refers to that lipid droplet-related triacylglycerols are hydrolysed by cytoplasmic lipases at pH 7. In contrast, acid lipolysis refers to that LD-related triacylglycerols are hydrolysed by lysosomal acid lipase at pH 4.5-5 [157].

Singh et al. [158] observed that inhibition of hepatic autophagy via 3-methyladenine (3-MA) treatment or Atg5 knockdown resulted in excessive accumulation of hepatic lipids and triglycerides in mouse liver. Furthermore, they investigated the rate of *β*-oxidation which is reflecting the level of FFA produced by triglyceride (TG) hydrolysis. The relative ratio of *β*-oxidation in Atg5-knockdown cells was significantly reduced compared with control cells. This result was consistent with the previous reduction in lipolysis. To further prove that autophagy regulated liver lipid metabolism, the authors measured the TG and cholesterol content in hepatocytes of mice with hepatocyte-specific Atg7 knockdown. They observed a significant increase in hepatic total cholesterol and TG accumulation. In contrast, the ratio of cholesterol in the lysosome was significantly reduced.

Subsequently, the role of lipophagy in promoting lipid metabolism was also confirmed in zebrafish liver cells. Wang et al. [159] observed the sequestered LDs in autophagic vacuoles of zebrafish liver cells by electron microscopy, thus confirming the occurrence of lipophagy. Inhibition of autophagy by chloroquine, a lysosomal acidification inhibitor that blocks the fusion of autophagosomes and lysosomes [160], significantly increased the LDs and TG content of the liver cells. Moreover, the chloroquine-induced lipophagy inhibition did also reduce the rate of *β*-oxidation significantly.

The decline of lipophagy in the aged or steatotic liver hinders the degradation of accumulated lipids in the liver and reduces the supply of FFAs for lipid metabolism, both further compromising cellular function [161, 162].

### 4.4. Impaired Mitophagy Leads to Decreased Mitochondrial Turnover and Increased ROS Production

Hepatocytes are rich in mitochondria which are the crucial organelles for lipid metabolism. Each hepatocyte includes about 800 mitochondria [163, 164]. Mitochondria act as the “energy plant” of the cells. Fatty acids can undergo *β*-oxidation to generate Coenzyme A (CoA), which enters the citric acid cycle (CAC) and produces abundant NADH and flavin adenine dinucleotide (FADH2). Both NADH and FADH2 enter the oxidative phosphorylation process and generate large amounts of ATP [163].

Ogrodnik et al. [165] observed that hepatocyte senescence caused mitochondrial dysfunction and impaired the capacity of fatty acid oxidation. This in return facilitated lipid accumulation and promoted age-related hepatic steatosis. Age-related mitochondrial dysfunction does not only affect lipid metabolism and ATP synthesis but also leads to the production of large amounts of reactive oxygen species (ROS).

Under normal physiological conditions, about 2% of oxygen is used for the production of reactive oxygen species [163, 166]. A basal level of ROS promotes cell survival and repair. However, high levels of ROS are detrimental, since they initiate fibrotic changes leading to structural impairment of the liver. For example, ROS and other lipid peroxidation products are activating hepatic stellate cells to produce extracellular matrix proteins ultimately contributing to the development of hepatic fibrosis. Besides, the increased ROS levels further aggravate the impairment of lipid metabolism finally resulting in hepatocyte apoptosis and hepatic inflammation [167–169].

**Figure 4:**
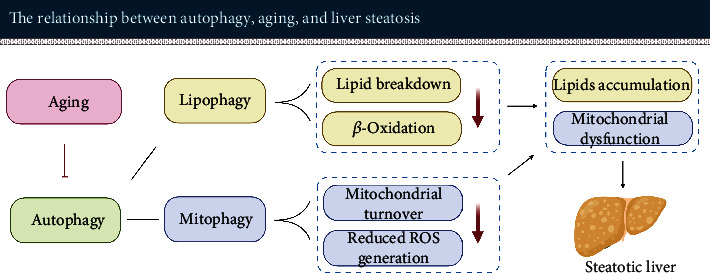
Aging-related decline of autophagy activity leads to impaired lipid metabolism in the liver. Impaired autophagy results in decreased lipid metabolism and in reduced mitochondrial turnover. These changes lead to the accumulation of lipid droplets in the hepatocytes and an increase of ROS production, which contributes to the accumulation of fat in the liver ultimately resulting in hepatic steatosis.

In brief, normal mitochondrial function is an important basis for maintaining hepatic metabolism. However, the age-associated impaired mitophagy in the liver leads to a decrease in mitochondrial turnover rate [170]. The number of dysfunctional mitochondria is increasing, which upregulates ROS production and ultimately aggravates hepatic steatosis (see Figure 4).

### 4.5. Restoring Autophagy Is Beneficial to Reduce Liver Steatosis

Numerous studies demonstrated that promotion of autophagy can effectively reduce lipid accumulation in the liver (see Table 1). Therefore, promoting autophagy may result in a novel therapeutic strategy to mitigate hepatic steatosis [158, 171, 172].

The following studies focused on inducing autophagy to reduce fat accumulation by using Resveratrol, Trehalose, and Catalpol, but also commonly known autophagy inducers such as rapamycin and carbamazepine.

Resveratrol is a natural polyphenol commonly found in grapes. Tang et al. [173] observed that Resveratrol treatment significantly enhanced the protein expression of LC3-II and Beclin-1, while p62 was reduced in C57BL/6J mice subjected to ethanol diet, indicating that autophagy was activated. In contrast, ethanol-induced steatosis was significantly alleviated in Resveratrol-treated mice, mainly manifested by a decrease of triglyceride, low density-lipoprotein cholesterol, and an increase of high-density lipoprotein cholesterol.

Trehalose is a natural disaccharide, which is usually used as a medical desiccant. Nowadays, it has attracted much attention as a mTOR-independent autophagy inducer [12, 174]. DeBosch et al. [175] observed that Trehalose prevented cells from taking up glucose via blocking glucose transporters in the plasma membrane. Doing so, Trehalose treatment induced a “starvation-like” condition triggering autophagy even in the presence of nutrients. Activation of autophagy alleviated accumulation of LDs in hepatocytes. This effect was attributed at least partly to preventing hexose uptake and subsequently triggering the AMPK-ULK1 pathway. Moreover, Trehalose significantly mitigated the accumulation of triglycerides induced by fructose in primary hepatocytes. Similar results were observed in independent experiments using the HepG2 cell line and mouse liver.

Catalpol is an iridoid glucoside mainly obtained from the root of Rehmannia glutinosa. This drug is commonly used for neurodegenerative diseases, e.g., Alzheimer's disease. Catalpol administration alleviated hepatic steatosis via enhancing autophagy in both ob/ob mice and mice subjected to a high-fat diet (HFD) as observed by Ren et al. [176]. They also reported that Catalpol mitigated Palmitate-induced lipid accumulation in HepG2 cells by activating autophagy via the AMPK-Transcription Factor EB (TFEB) pathway. In contrast, treatment with the AMPK inhibitor (Compound C) almost abolished the protective effect of Catalpol on lipid accumulation in HepG2 cells, supporting the crucial role of autophagy in hepatic steatosis.

Using other autophagy inducers as done by Lin et al. [177] resulted in similar observations: they reported that rapamycin and carbamazepine also relieved hepatic steatosis in C57BL/6 mice by inducing autophagy. In contrast, treatment of mice with autophagy inhibitors (chloroquine) exacerbated hepatic steatosis and injury.

To our knowledge, there is no widely accepted pharmacological strategy for fatty liver disease. Many clinical guidelines recommended that exercise is an effective way to improve nonalcoholic fatty liver disease. Recent studies demonstrated that exercise may improve NAFLD through enhancing autophagy as well. Chun et al. [178] reported that exercise may trigger hepatic autophagy via regulating muscle-derived myokines. First, postexercise reduction of C1q/TNF-related protein 5 (CTRP5) inhibited the activity of the mTORC1 to induce autophagy. Second, the increase of irisin, a myokine secreted by skeletal muscle after exercise, promoted the stimulation of AMPK. The subsequent activation of AMPK activated ULK1 resulting in enhanced autophagy. Besides, exercise can also induce autophagy via releasing Beclin-1 from its complex with B-cell lymphoma-2 (Bcl2). As mentioned before, Beclin-1 can promote autophagy via forming a PI3K-3 complex which is crucial for the initiation of autophagosomes [133].

To sum up, activating autophagy removes dysfunctional mitochondria, reduces ROS production, degrades excess lipids, and promotes *β*-oxidation in the steatotic liver. Therefore, modulating autophagy seems to be an effective strategy in alleviating liver steatosis and preventing the development of fatty liver diseases, possibly also suitable to treat age-related steatosis.

## 5. Liver Fibrosis

Liver fibrosis is the consequence of an imbalance in the generation and degradation of extracellular matrix (ECM), which is usually caused by acute or chronic liver damage [189]. In essence, liver fibrosis is a wound healing response to various liver injuries. Aging is considered as one of the important risk factors for liver fibrosis [190]. Liver fibrosis may gradually progress to liver cirrhosis in case of chronic liver damage. At present, there is no effective clinical treatment for liver fibrosis.

Hepatic stellate cells (HSCs) reside in the space of Disse and account for about 5-8% of the total number of liver cells. In a normal liver, most of the HSCs are at a quiescent state with low proliferative activity [191, 192]. Activation of hepatic stellate cells (HSCs) is now widely recognized as a major driver for the initiation and progression of hepatic fibrosis in rodents and humans [193]. Hepatic stellate cells are usually activated when the liver undergoes injury. Activated HSCs are characterized by increasing proliferation, chemotaxis, and contractility. Upon activation, they secrete large amounts of fibrogenic factors that facilitate the generation of collagen. Excessive deposition of the extracellular matrix is indicative of hepatic fibrosis [194–197].

Quiescent HSCs contain high amounts of cytoplasmic LDs with triglyceride and retinyl esters. During the process of HSC activation, LDs are degraded and activate HSCs to secrete excessive amounts of the extracellular matrix proteins such as collagen and fibronectin. Upon activation, HSCs undergo a transformation from LD-rich cells to myofibroblast-like cells, a process which is accompanied by an upregulation of autophagic flux [198–200].

### 5.1. Autophagy Provides Energy for Activation of Hepatic Stellate Cells via Lipid Degradation

Autophagy may provide energy to promote the activation of HSCs [198–200]. This view is supported by several independent authors.

Hepatic injury triggers autophagy which in turn promoted ATP-production. Hernández-Gea et al. [201] observed that hepatic injury induced by carbon tetrachloride (CCl_4_) or thioacetamide (TAA) enhanced the autophagy level in C57BL/6 mice. They established HSC-specific Atg7-knockdown mice. After inducing chronic fibrosis using CCl_4_ for 6 weeks in genetically modified and wild-type mice, the collagen accumulation in Atg7-knockdown mice was significantly decreased compared with control mice. Interestingly, the number of *α*-SMA positive HSCs in Atg7-knockdown animals was not significantly different from that in control mice. However, the expression of total *α*-SMA protein in HSCs of Atg7-knockdown animals was significantly reduced, indicating that the absence of Atg7 reduced the expression of *α*-SMA in each HSC instead of affecting the number of HSCs. In addition, Atg5/7 knockdown, as well as pharmacological inhibition of autophagy through administration (3-MA or chloroquine), substantially reduced fibrogenic mediators in mouse stellate cells. It is worth noting that there was a significant increase in the number of LDs in mouse stellate cells obtained after Atg5/7 knockdown, respectively, 3-MA treatment.

Furthermore, 3-MA-mediated inhibition of autophagy caused a substantial decrease in ATP levels of the cells. In contrast, the administration of oleic acid in mouse stellate cells enhanced ATP levels and abolished the reduction of fibrogenesis mediated via inhibition of autophagy. These results imply that autophagy facilitated the breakdown of lipid droplets into FFAs in mouse stellate cells. Subsequently, these FFAs are oxidized in mitochondria to generate ATP needed for the activation of mouse stellate cells.

Moreover, Thoen et al. [198] found a significant elevation in autophagy levels during HSC activation. In contrast, inhibition of autophagy prevented HSC activation. In more detail, they treated HSCs with Bafilomycin A1, a V-ATPase inhibitor preventing the acidification of lysosome and the fusion of the autophagosome with lysosome [202, 203]. Bafilomycin A1 treatment of HSCs resulted in a significant decrease in *α*-SMA. The proliferation rate of Bafilomycin A1-treated HSCs was reduced by approximately 6-fold compared to control HSCs. In contrast, HSCs responded to activation when Bafilomycin A1 treatment was discontinued.

Inhibition of autophagy using chloroquine resulted in similar observations. He et al. [204] demonstrated that chloroquine attenuated CCl_4_-induced liver fibrosis in Sprague-Dawley rats by inhibiting autophagy and thereby HSC activation. Compared with the animals of the control group, the expression levels of serum ALT, aspartate aminotransferase (AST), hydroxyproline (an extracellular matrix marker), and *α*-SMA were significantly decreased in the chloroquine group. In contrast to Hernández-Gea et al.'s findings, He et al. observed that chloroquine-mediated autophagy inhibition improved liver injury as well. This additional finding may be related to the fact that they did not only use a different modeling method but also a different species of animals.

Taken together, these studies imply that induction of autophagy promotes the initiation of liver fibrosis by degrading intracellular lipids to provide the energy needed for HSC activation.

However, rapamycin, an autophagy inducer known for its antiproliferative effect, had an opposite effect and reduced hepatic fibrosis. In this case, the effect was attributed to the antiproliferative effect on HSCs rather than to the autophagy-inducing capacity suggesting the promotion of fibrosis. For better illustration of this seemingly contradictory effect, we describe the experimental observations reported by Zhu et al. [205]. They investigated the effect of rapamycin on hepatic stellate cells in Sprague-Dawley rats with CCl_4_-induced liver fibrosis. In their hands, rapamycin treatment reduced the extent of rat liver fibrosis induced by CCl_4_ compared to the control group.

Further experiments revealed that rapamycin significantly inhibited the proliferation of HSCs stimulated by the platelet-derived growth factor. However, treating HSCs with rapamycin did not significantly affect the expression of ECM-related proteins. The inhibition of HSC proliferation by rapamycin appeared to be the main reason for its alleviation of liver fibrosis. This antiproliferative effect of rapamycin on various cell types has been confirmed repeatedly in different studies [206–208].

We will elucidate the mechanism conveying its antiproliferative effect in the next section dedicated to explain the impact of aging and autophagy on liver regeneration later in this review (Section 6.4).

### 5.2. Autophagy May Indirectly Reduce Fibrosis by Ameliorating Liver Injury

Hepatic fibrosis is a common result of different liver diseases such as NAFLD, alcoholic hepatitis, and drug intoxication. Induction of autophagy is currently considered to exert a therapeutic effect on these hepatic diseases causing liver injury. For example, alcohol abuse increases liver metabolic burden, induces accumulation of lipid droplets, and impairs mitochondrial function leading to increased oxidative stress. Autophagy may alleviate alcohol-induced hepatic injury by selectively eliminating dysfunctional mitochondria (mitophagy) and lipid droplets (lipophagy) [209].

Moreover, intoxication with acetaminophen, a commonly antipyretic drug, can cause severe liver damage such as acute hepatocyte necrosis and mitochondrial damage [210]. Ni et al. [210] observed that rapamycin-induced autophagy mitigated acetaminophen-induced hepatotoxicity via eliminating impaired mitochondria in C57BL/6 mice. In contrast, inhibition of autophagy using chloroquine aggravated acetaminophen-induced hepatotoxicity.

### 5.3. The Effect of Modulating Autophagy on Liver Fibrosis Is Dependent on Cell Types

**Figure 5:**
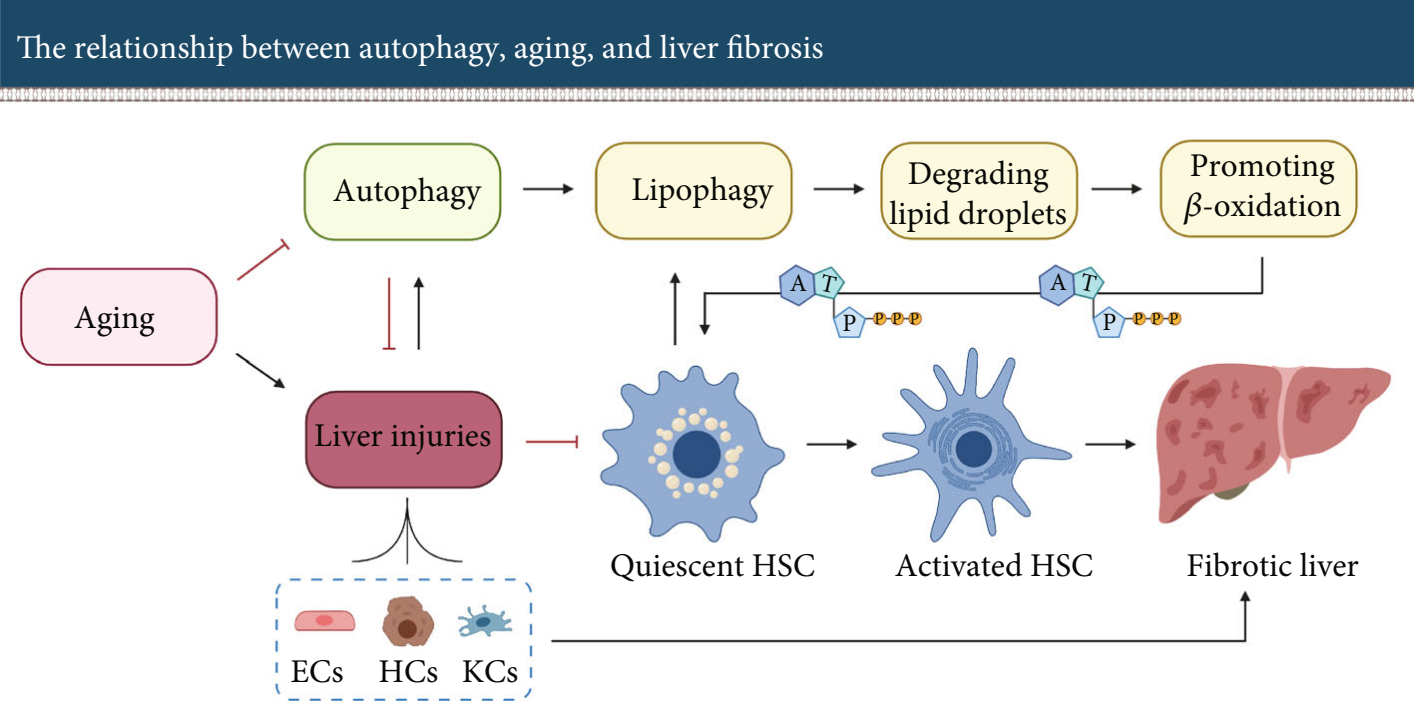
Autophagy plays a dual role in liver fibrosis. First, it promotes the initiation of fibrosis by providing the energy required for activation of hepatic stellate cells. On the other hand, it alleviates liver fibrosis by improving the function and status of other hepatic cells such as hepatocytes, endotheliocytes, and macrophages. ECs: endotheliocytes; HCs: hepatocytes; KCs: Kupffer cells.

Inhibition of autophagy may mitigate hepatic fibrosis by alleviating hepatocyte injury, by reducing endothelial dysfunction, and by decreasing inflammatory cytokines synthesized and released from macrophages (see Figure 5) [211–214].

Here, we present the experiments of Lodder et al. [213] for further illustration that autophagy inhibition via Atg5-knockout aggravated fibrosis. Lodder et al. stated that macrophages were involved in promoting both inflammatory and liver fibrogenesis by secreting cytokines such as ROS-induced IL-1A/B. Compared with wild-type mice, Atg5-knockdown mice subjected to treatment with CCl_4_ demonstrated higher levels of proinflammatory cytokines IL-1A/B in the liver. Furthermore, mice with Atg5-knockdown developed a higher degree of fibrosis compared to wild-type animals. These mice also showed higher protein level of fibrosis-related proteins such as *α*-SMA and mRNA expression of fibrogenic-related genes such as matrix metallopeptidase 9 (Mmp9), transforming growth factor beta 1 (TGF-*β*1), and serpine 1 in the liver. Administration of the Atg5-knockout mice with recombinant interleukin-1 receptor antagonist (IL-1RN) substantially reduced CCl_4_-induced liver injury and fibrosis. Taken together, these results illustrate that autophagy attenuates liver fibrosis by reducing the release of IL-1A/B.

Similarly, Ruart et al. [214] demonstrated that selective autophagy suppression by cell-specific Atg7 knockdown in endothelial cells exacerbated CCl_4_-induced liver fibrosis in mice. Autophagy suppression decreased the ability of LSECs to respond to oxidative stress and led to endothelial dysfunction, which in turn activated HSCs. The authors observed a marked reduction in the porosity and number of fenestrae in LSECs of Atg7-knockdown mice via scanning electron microscopy. Besides, hydroxyproline and *α*-SMA expression in mouse liver was increased, but there was no difference in the expression of platelet-derived growth factor receptor beta (PDGFR-*β*, a proliferation marker of HSCs). These results reflected that the aggravation of liver fibrosis in Atg7-knockdown mice may be due to EC-mediated activation rather than proliferation of HSCs.

### 5.4. Selective Inhibition of Autophagy in HSC Appears to Be a Promising Antifibrosis Strategy for the Aging Liver

As explained above, autophagy has a dual role in the process of liver fibrosis (see Table 2). On the one hand, upregulation of autophagy induces HSC activation, leading to the initiation and progression of hepatic fibrosis. On the other hand, upregulation of autophagy may also result in an antifibrotic effect. However, the profibrotic effect of inducing autophagy and thereby providing energy for HSC activation seems to be more pronounced than the antifibrotic effect exerted by relieving cellular oxidative stress and inflammation.

It is worth noting that basal autophagy takes place continuously in eukaryotes as it is essential for intracellular homeostasis and cellular self-renewal [215, 216]. As the autophagy activity in the aged liver declines, further inhibition of autophagy may cause serious adverse effects for the liver and other organs. Therefore, only selective inhibition of autophagy in HSCs appears to be a potentially effective antifibrotic strategy.

## 6. Impaired Liver Regeneration

Unlike other visceral organs, the liver has an amazing capacity for regeneration. It is the pathophysiological basis for successful surgery such as liver resection and partial liver transplantation. After rodents undergo 2/3 partial hepatectomy (PH), the remaining liver tissue is almost restored to its original volume and function in about 1-2 weeks [225, 226].

### 6.1. Liver Regeneration Is Mainly Accomplished by Two Different Regenerative Mechanisms

Liver regeneration is mainly achieved by two regenerative mechanisms: first, the division of mature hepatocytes; second, the renewal and differentiation of liver progenitor cells (LPCs) [12, 227].

The first mechanism of liver regeneration consists of well-orchestrated hepatocyte proliferation, a sophisticated process that includes three phases: the priming stage, proliferation stage, and termination stage. In the priming stage, quiescent hepatocytes shift from G0 to G1 phase within 4 hours after PH-induced stimulation in rodents [228]. In the proliferation stage, hepatocytes are stimulated by several mitogens such as hepatocyte growth factor (HGF) and transforming growth factor-alpha (TGF-*α*) to cross the restriction point of the G1 phase. Then, they enter the synthesis and mitotic phase [229–234]. The termination stage starts once liver mass is almost restored to its original level. Hepatocyte proliferation ceases under the regulation of transforming growth factor beta (TGF-*β*), activin, and interleukin-1A/B (IL-1A/B) [226, 235–245].

The second mechanism is based on LPCs, which are involved in the regeneration of animal livers under certain conditions [246, 247]. LPCs are bipotent progenitor cells that reside in the canal of Hering. When the liver is severely injured or is chronically damaged, the remaining hepatocytes may not be able to meet the regenerative demand. Then, LPCs will be activated and promote liver regeneration via renewing and differentiating into hepatocytes and cholangiocytes [248–251].

### 6.2. Aging Significantly Impairs Liver Regeneration

Aging leads to a significant decrease in the regenerative capacity of the liver in respect to hepatocyte proliferation as well as LPC division and differentiation. In the aged liver, there are fewer hepatocytes entering the S phase (about 30%) compared with the young liver (90%-100%). Furthermore, senescent hepatocytes enter the S phase more slowly [252].

Also, the responsiveness of LPCs to liver injury decreases with age. For example, Cheng et al. reported that LPCs of young mice are activated to proliferate following chronic liver injury induced by a choline-deficient, ethionine-supplemented (CDE) diet. However, LPCs in aged mice did not respond effectively to the injury, leading to defective liver regeneration. According to Cheng et al., hepatic stellate cells of aged mice secreted more chemokine (C-X-C motif) ligand 7 (CXCL7) than those of young mice, attracting more neutrophils to infiltrate the liver. Neutrophil infiltration resulted in excessive ROS production, thereby restraining the activation and proliferation of LPCs, which further impaired liver regeneration [247].

As described before, aging causes structural changes of the liver such as steatosis and fibrosis. Both can be further aggravated by the lifestyle of the patients, e.g., dietary overload or extensive alcohol consumption leading to NAFLD and/or alcoholic fibrosis and even cirrhosis. Both NAFLD and alcoholic cirrhosis are further impairing liver regeneration substantially [253, 254]. The overall impaired regenerative capacity of the aged liver leads to a remarkably increased risk of hepatic failure after partial hepatectomy [255].

### 6.3. Autophagy Provides the Necessary Energy for Liver Regeneration

Liver regeneration is an energy-intensive process. The division and growth of hepatocytes require abundant energy supply [256]. Hepatocytes are rich in mitochondria, but liver resection can cause substantial mitochondrial damage and decrease hepatocyte ATP synthesis. Correspondingly, Toshima et al. reported a significant decrease in ATP reserves within 6 h after liver resection [257].

**Figure 6:**
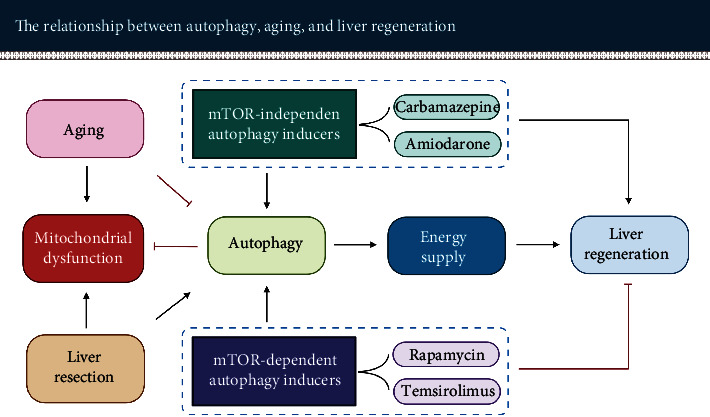
The effect of autophagy on liver regeneration in the aged individual. Liver resection and aging lead to mitochondrial dysfunction. Autophagy can degrade dysfunctional mitochondria and other cellular components to promote the synthesis of new organelles and energy production, thereby facilitating liver regeneration. However, the induction of autophagy through the mTOR pathway impedes cell proliferation. Therefore, inducing autophagy via the mTOR-independent pathway is more appropriate for promoting liver regeneration.

During the initial stage of liver regeneration, autophagy, particularly mitophagy, is crucial for maintaining healthy mitochondria to generate ATP. Mitophagy can selectively eliminate dysfunctional mitochondria in order to reduce ROS production, promote mitochondrial regeneration, and facilitate ATP synthesis [62–64, 215]. This process contributes to the required energy and environment for liver regeneration (see Figure 6). However, the autophagy level in the aged liver is significantly reduced. Therefore, appropriate induction of autophagy seems to be a promising strategy to promote regeneration, especially of the aged liver.

### 6.4. Autophagy Induced through the mTOR-Dependent Pathway Impairs Liver Regeneration

As mentioned above, autophagy can be activated by both mTOR-dependent pathways and mTOR-independent pathways. However, mTOR is not only an essential regulator of autophagy but also a key regulator of cell proliferation [12, 98]. Inhibiting mTOR induces autophagy, but it also significantly impairs cell proliferation.

For example, rapamycin, a classic mTOR inhibitor, induces autophagy by inhibiting mTOR activity. Rapamycin inhibits mTORC1 by forming a complex with FK506-binding protein 12. This complex acts on downstream targets to restrain protein synthesis and causes cell-cycle arrest by preventing the transition from G1 to S phase [87, 258]. The antiproliferative effect has been demonstrated in several independent experiments regarding liver regeneration [208, 259]. Similar results have also been observed with other mTOR inhibitors such as Temsirolimus [260]. Therefore, activating autophagy by inhibiting mTOR activity does not seem to be appropriate for facilitating liver regeneration (see Figure 6).

### 6.5. Autophagy Induced through the mTOR-Independent Pathway Appears to Promote Liver Regeneration

Activation of autophagy without suppression of cell proliferation is a better option for liver regeneration. Therefore, inducing autophagy via the mTOR-independent pathway seems promising in promoting liver regeneration (for more molecular details, see also Xu et al. [12]). By now, the role of this pathway for liver regeneration has been investigated by a number of authors (see Table 3). They demonstrated that the use of different mTOR-independent autophagy inducers such as carbamazepine and amiodarone promoted liver regeneration.

Carbamazepine is a common antiepileptic medication that can be used to prevent and control seizures. It has recently been shown to induce autophagy through depletion of cytosolic inositol and AMPK activation. The depletion of cytosolic inositol causes a decrease in basal IP_3_, which reduces energy production via blocking mitochondrial calcium influx. The reduced energy level activates the AMPK-ULK1 pathway to enhance autophagy [261, 262].

In 2013, Kawaguchi et al. [260] observed that carbamazepine treatment substantially promoted hepatocyte proliferation after PH in mice through activation of mTOR and its downstream factor S6K. Three proliferation indices, Ki-67, 5-Bromo-2′-Deoxyuridine (BrdU), and the Proliferating Cell Nuclear Antigen (PCNA) index as well the LBWR levels, were significantly increased on postoperative day 2 (POD2) in carbamazepine-treated animals compared to control animals. On the contrary, the application of mTOR inhibitor Temsirolimus abolished the effect of carbamazepine in promoting hepatocyte proliferation, indicated by a marked decrease in the protein expression of PCNA and LBWR of animals on POD2.

Amiodarone is a potent antiarrhythmic medication that is mainly used to promote the restoration of normal heart rhythm. It is currently obtaining attention as an autophagy inducer. Amiodarone treatment decreases intracellular Ca^2+^ concentration by inhibiting L-type Ca^2+^ channels at the plasma membrane to block extracellular Ca^2+^ entry. Reducing intracellular Ca^2+^ concentration can induce autophagy [132, 263, 264]. In 2015, Lin et al. [265] observed that amiodarone could significantly induce autophagy via the mTOR-independent pathway and boost liver regeneration. After PH, LC3-II was significantly higher and p62 level lower in the amiodarone-treated mice compared to the control mice. The Ki-67, PCNA, cyclin D1 levels, and LBWR were substantially increased, but the level of p21 decreased significantly in amiodarone-treated mice, altogether demonstrating an improved hepatic proliferative response. As a contrast, inhibition of autophagy via chloroquine pretreatment or Atg7 knockdown deteriorated liver regeneration. Correspondingly, decreased Ki-67, PCNA, cyclin D1, and LBWR and increased TGF-*β*1 were observed in the autophagy-suppressed mice.

Overall, selecting the appropriate pathway to induce autophagy is essential for promoting liver regeneration. Enhancing autophagy through the mTOR-dependent pathway alone seems to be rather harmful to liver regeneration. In contrast, inducing autophagy through the mTOR-independent pathway does not affect cell proliferation. Therefore, exploring novel mTOR-independent autophagy inducers without obvious side effects has the great potential to improve liver regeneration, especially in aged patients with reduced autophagy.

## 7. Mitochondrial Dysfunction

Mitochondria are the sites of oxidative phosphorylation in cells [270]. The synthesis of ATP through oxidative phosphorylation is one of the key functions of mitochondria. This process is regulated by four respiratory chain complexes, type I NADH dehydrogenase (complex I), succinate dehydrogenase (complex II), CoQH2-cytochrome c reductase (complex III), cytochrome c oxidase (complex IV), and another ATP synthase (complex V). All these complexes are located on the inner membrane of mitochondria [271–273]. Mitochondrial bioenergy is pivotal to maintain liver function. Mitochondrial dysfunction can lead to impaired energy metabolism and increased production of reactive oxygen species, which in turn triggers cell senescence and apoptosis [20, 274].

### 7.1. Aging Impairs the Function of Hepatic Mitochondria

Evidence in human and animal liver manifests that aging results in increased oxidative stress and decreased mitochondrial bioenergetics. Actually, mitochondrial dysfunction is considered to be one of the crucial features of the aging process.

One feature of mitochondrial dysfunction is the loss of activity of mitochondrial enzymes. To give one example, Navarro and Boveris [275] observed in aged rat livers that the activity of key enzymes indicative of mitochondrial function decreased substantially compared to young rats. They investigated type I NADH dehydrogenase (complex I), cytochrome oxidase (complex IV), mitochondrial nitric oxide synthase, and Mn-superoxide dismutase and reported a loss of activity in these enzymes of about 30%, 24%, 47%, and 46%, respectively. The reduced activity of respiratory chain complexes impaired energy synthesis of mitochondria in the hepatocytes from aged rats. Yen et al. [276] used human livers and confirmed that mitochondrial respiration was also deficient in isolated mitochondria from the aging human liver.

Other features of mitochondrial dysfunction are the reduction of the mitochondrial membrane potential (∆*Ψ*m) and the increase of peroxide production. Sastre et al. [277] used liver cells from aged Wistar rats in comparison to young rats and observed a 30% reduction in mitochondrial membrane potential and a 23% increase in mitochondrial peroxide production. This was accompanied by an age-related increase in size.

#### 7.1.1. Age-Related Mitochondrial Dysfunction Is Associated with the Accumulation of mtDNA Mutations

Mitochondria contain their own genome, a 16.5 kb double-stranded circular molecule (mtDNA) which encodes 2 mammalian ribosomal RNAs, 22 transfer RNAs, and 13 proteins. The 13 proteins encoded by mtDNA are the constituent of respiratory chain enzymes [273, 278]. Mitochondrial dysfunction is usually associated with mitochondrial DNA (mtDNA) mutations.

mtDNA is located near the main site of ROS generation—the respiratory chain. ROS are a byproduct of oxidative metabolism. It can induce oxidative damage to mtDNA and is thought to be responsible for mtDNA mutations that accumulate with aging [279, 280]. Vermulst et al. [281] established a genetically modified animal that enhances the expression of human catalase (a ROS scavenger). The level of mtDNA mutations in heart and mouse embryonic fibroblasts (MEFs) of these animals was significantly lower than in WT animals. This result demonstrated that oxidative stress plays a negative role in mtDNA mutations. The cumulative effect of ROS affects genetic information of mtDNA causing point mutations, deletions, or duplications of mtDNA [282]. Ultimately, the accumulation of mtDNA mutations and ROS leads to impaired respiratory chain activity and energy production [283].

In 2004, Trifunovic et al. [284] established mtDNA mutator mice that express a checking-deficient version of PolgA (mtDNA polymerase gamma). The mtDNA mutator mice successfully express a mtDNA mutant phenotype with an about 4-fold increase in point mutations in the liver and a concomitant increase of deleted mtDNAs. The lifespan of mtDNA mutant mice was significantly shorter compared to control animals. Phenotypic features related to aging such as fertility did decline. In contrast, age-related impairments like osteoporosis and anaemia did appear prematurely in these animals. Moreover, the enzymatic activity of the respiratory chain was decreased. These results suggest that mtDNA mutations cause mitochondrial dysfunction and aggravate the aging process.

But this view has also been challenged. In 2007, Vermulst et al. [281] could not confirm that mtDNA mutations shortened the longevity of wild-type mice. Although the point mutations of mtDNA in wild-type mice increased about 11-fold with age, mitochondrial mutator mice could tolerate a 500-fold higher mutational burden than control mice without any evident accelerated aging characteristics. It is worth noting the authors pointed out that their technique can only detect small deletion of mtDNA but not the large-scale deletion of mtDNA.

Overall, the age-related ROS increase is considered to be an important causal factor of mtDNA mutations. mtDNA mutations substantially impair the efficiency of the respiratory chain and contribute to mitochondrial dysfunction. However, whether mtDNA mutations directly accelerate the aging process and affect human lifespan still needs further investigation.

#### 7.1.2. Aging Impairs Mitochondrial Dynamics

**Figure 7:**
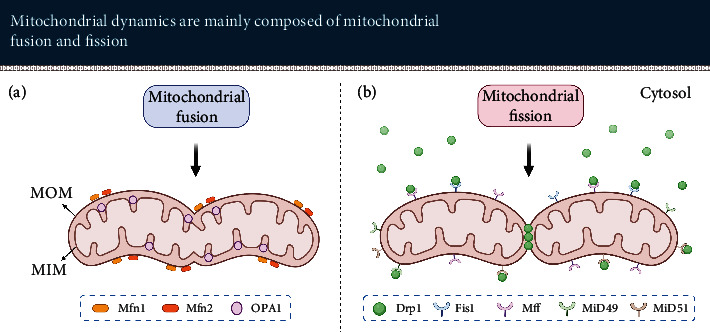
Mitochondrial dynamics mainly include mitochondrial fusion and fission. Mitochondria perform fusion and fission under the joint regulation of multiple signals. (a) Mfn1 and Mfn2 regulate mitochondrial outer membrane fusion; OPA1 regulates mitochondrial inner membrane fusion. (b) Fis1, Mff, MiD49, and MiD51 are anchored at the mitochondrial outer membrane to recruit Drp1 from the cytosol, which facilitates that mitochondria contract and split into several mitochondria. MOM: mitochondrial outer membrane; MIM: mitochondrial inner membrane.

Mitochondria are dynamic organelles that constantly undergo fission and fusion to form network structures in cells. This process usually is termed mitochondrial dynamics (see Figure 7) [285, 286]. It is involved in regulating the morphology, distribution, and property of mitochondria [285, 287, 288].

Mammalian mitochondrial fusion is mainly mediated by mitofusin 1 (Mfn1), Mfn2, and optic atrophy 1 (OPA1). All of them are dynamin-related GTPases, but their function is different during mitochondrial dynamics. Mfn1 and Mfn2 are involved in fusing the outer membranes of mitochondria, while OPA1 is in charge of fusing the inner membranes of mitochondria. Mammalian mitochondrial fission is mainly regulated by dynamin-related protein 1 (Drp1). It interacts with his receptor proteins, mitochondrial dynamics protein of 49 kDa (MiD49), MiD51, fission 1 (Fis1), and mitochondrial fission factor (Mff), to promote the constriction of mitochondrial membrane and mitochondrial fission [285, 289].

Mitochondrial dynamics plays a vital role in mitochondrial quality control. Malfunctioning mitochondria may lose their fusing capacity to prevent damaged mitochondria from merging back into the mitochondrial network [289]. These dysfunctional mitochondria will be degraded by mitophagy. However, the age-dependent decline of mitophagy not only inhibits the clearance of dysfunctional mitochondria but also affects the mitochondrial biogenesis, leading to the gradual accumulation of dysfunctional mitochondria [290].

### 7.2. Mitophagy Effectively Promotes Mitochondrial Turnover

Dysfunctional mitochondria promote ROS generation. Mitophagy can selectively degrade damaged mitochondria, reduce excessively produced ROS, facilitate mitochondrial regeneration, and promote the survival of cells in stressful environments [291, 292]. The serine/threonine kinase PTEN-induced kinase 1(PINK1) and E3 ubiquitin ligase Parkin are considered to be two crucial factors that mediate mitophagy.

PINK1 is thought to sense mitochondrial quality. It includes a mitochondrial targeting sequence (MTS) and can be recruited into mitochondria. In normal mitochondria, PINK1 is translocated into the outer mitochondrial membrane through the translocase outer membrane (TOM) complex and into the inner mitochondrial membrane with the mediation of the translocase inner membrane (TIM) complex. The MTS fragment of PINK1 is cleaved by the mitochondrial processing peptidase (MPP) in the matrix. Then, PINK1 is degraded by the proteasome system controlled by presenilin-associated rhomboid-like protease (PARL). This process regulates the concentration of PINK1 in normal mitochondria.

**Figure 8:**
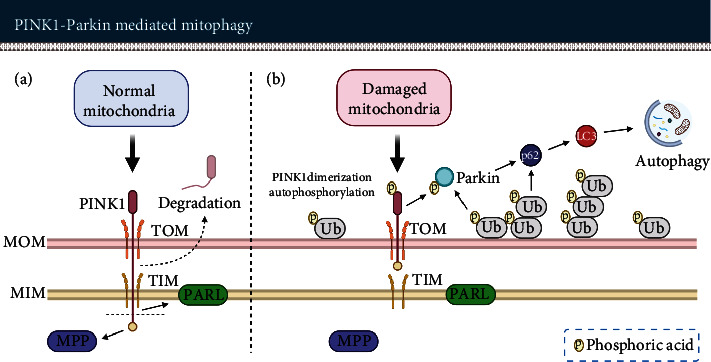
PINK1-Parkin-mediated mitochondrial autophagy (mitophagy) eliminates damaged mitochondria. (a) In the case of normal mitochondria, Pink1 is cleaved twice by MPP and PARL then decomposed by the proteasome system. (b) When mitochondria are damaged, due to changes such as the dissipation of membrane potential (∆*Ψ*m), PINK1 aggregates in the outer mitochondrial membrane to undergo dimerization and autophosphorylation. Parkin and ubiquitin are phosphorylated via PINK1. PINK1-Parkin facilitates mitophagy via the recruitment of autophagy receptor proteins and mitochondrial ubiquitination. MOM: mitochondrial outer membrane; MIM: mitochondrial inner membrane; TOM: translocase outer membrane; TIM: translocase inner membrane.

In damaged mitochondria, mitochondria depolarize due to various injuries. This membrane potential is crucial for TIM-mediated protein translocation. Based on that, most of PINK1 is unable to enter the inner membrane and cannot be degraded by PARL-mediated degradation. In consequence, PINK1 accumulates on the outer mitochondrial membrane and phosphorylates ubiquitin. Then, accumulated PINK1 is activated via dimerization and autophosphorylation. Autophosphorylation of the PINK1 at S228 and 402 sites occurs after mitochondrial depolarization, which is thought to be a precondition for recruiting Parkin. PINK1 phosphorylates Parkin at the S65 site. PINK1/Parkin triggers autophagy via recruitment of the autophagic-substrate proteins such as p62 and mitochondrial ubiquitination [65, 289, 293–298] to eliminate damaged mitochondria (see Figure 8).

### 7.3. Enhancing Autophagy Is a Promising Way to Improve Mitochondrial Function in the Aged Liver

Recent studies revealed a number of interesting approaches in different model organisms (see Table 4) suitable to improve mitochondrial function via enhancing autophagy. Here, we are presenting the results of pharmacological upregulation using quercetin, melatonin, urolithin 1, and tomatidine, and via mTOR knockdown. Interestingly, they also improved the longevity of the model organisms.

Quercetin administration upregulates mitophagy thereby effectively reducing the impact of mitochondrial injuries as reported by Yu et al. [299]. They observed that chronic ethanol diet administration caused significant damage to hepatic mitochondria of C57BL/6J mice. Mitochondrial injury mainly manifested as mitochondrial swelling, internal membrane destruction, lack of cristae, rupture of the endoplasmic reticulum, and decrease of mitochondrial membrane potential. Quercetin administration effectively reduced these mitochondrial injuries by activating mitophagy. The mRNA and protein expression of Parkin was significantly decreased in the ethanol diet administration mice compared with control mice, while Parkin expression was significantly increased after quercetin coadministration. These results reflect that mitophagy activation exerts a crucial role in improving hepatic mitochondrial dysfunction.

Melatonin, a hormone that is usually used to enhance sleep quality, improved hepatic mitochondria function by activating mitophagy as reported by Zhou et al. [300]. They found that the protein expression of mitochondrial-LC3-II, Atg5, and Beclin-1 in mouse primary hepatocytes was substantially decreased after treatment with palmitic acid but significantly increased after treatment with melatonin. Palmitic acid caused mitochondrial damage indicated by a reduced oxygen consumption rate, decreased ATP synthesis, and dissipation of mitochondrial membrane potential, while melatonin effectively alleviated the above mitochondrial dysfunctions.

Urolithin A, a bacterial metabolite of ellagic acids [301], could trigger mitophagy in vivo and in vitro, as observed by Ryu et al. [302, 303]. Urolithin A prevented the age-related accumulation of damaged mitochondria in C. elegans, a model organism used frequently in aging research, and prolonged their lifespan. In mammalian cells, urolithin A was able to induce mitophagy and lead to an increase of phospho-AMPK*α*. Furthermore, in rodents, urolithin A promoted mitophagy leading to improved mitochondrial biogenesis and mitochondrial function, which was indicated by the enhanced aerobic endurance and grip strength of the animals.

Similarly, tomatidine, a steroidal alkaloid from unripe tomato [304], did enhance longevity in C. elegans by inducing mitophagy, as reported by Fang et al. [305]. Tomatidine sustained mitochondrial homeostasis via regulating PINK-1/DCT-1-dependent mitophagy and mitochondrial biogenesis. Besides, tomatidine could effectively relieve age-related changes in C. elegans. For example, tomatidine substantially improved the decline in age-related swimming scores in aged C. elegans. Compared with the vehicle group, the score increased by 48%.

Reducing mTOR expression via mTOR knockdown in mice as performed by Wu et al. [306] prolonged the overall lifespan by 20% compared to control animals. The mRNA expression of p16 in the liver of aged mTOR-knockdown mice was significantly lower than that of control animals.

These results from strikingly different experiments all suggest that upregulation of autophagy seems to be effective to alleviate age-related impairment.

## 8. Conclusion

**Figure 9:**
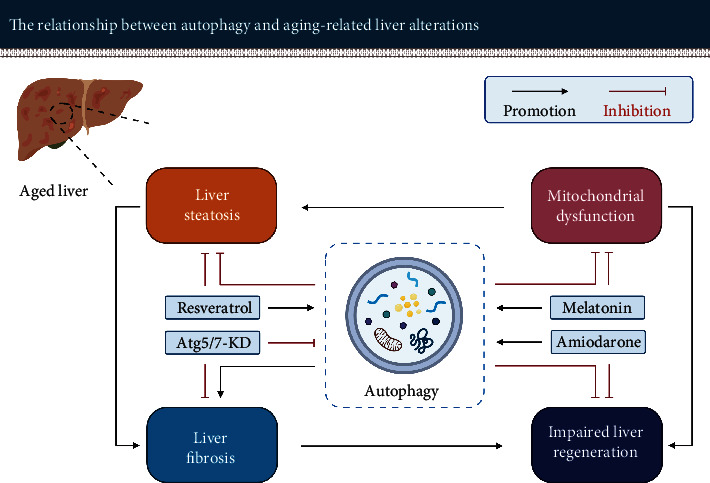
Autophagy plays different roles in age-related liver alterations. Enhancing autophagy may ameliorate aging-induced liver steatosis, mitochondrial dysfunction, and the impaired regenerative capacity but may aggravate liver fibrosis injury. KD: knockdown.

Aging is a natural phenomenon that occurs in all eukaryotic organisms. The aging process predisposes the liver to certain histopathological lesions, to decreased metabolic function, and to an impaired regenerative capacity. Accumulating evidence suggests that autophagy is involved in a variety of physiological and pathological events in the liver. Of concern is that modulation of autophagy has different effects on aging-induced changes in the liver (see Figure 9).

*For liver steatosis*: an appropriate boost in autophagy can effectively promote lipid metabolism and reduce lipid accumulation in hepatocytes. Inducing autophagy, e.g., Resveratrol may contribute to relieving the metabolic burden of the aging liver as well as prevent or slow down the initiation and progression of NAFLD, especially in elderly patients with impaired autophagy.

*For liver fibrosis*: upregulation of autophagy appears to provide the energy required for activation of hepatic stellate cells in case of hepatic injury. However, the induction of autophagy is also thought to be beneficial in reducing liver cell injury. Reduction of liver cell injury may improve fibrosis, which is relatively limited compared to the direct profibrosis effect. Therefore, selective inhibition of autophagy in hepatic stellate cells using, e.g., Atg5/7 knockdown seems to be a promising experimental strategy to counteract liver fibrosis in aged livers.

*For impaired liver regenerative capacity*: enhancement of autophagy via the mTOR-independent pathway, e.g., amiodarone, seems to be helpful. In this case, cell proliferation is not affected but the energy required for hepatocyte division and growth provided, thereby promoting liver regeneration. This is of utmost benefit for elderly patients who desperately need a life-saving liver resection.

*For mitochondrial dysfunction*: activation of autophagy can effectively eliminate dysfunctional mitochondria and promote mitochondrial regeneration. Both are of equal importance for reducing ROS and facilitating hepatocyte survival. Adequate and healthy mitochondria in turn facilitate the breakdown of hepatic lipids and provide energy to maintain liver function. Prevention or reversing mitochondrial dysfunction by inducing autophagy, e.g., melatonin, could be a promising therapeutic approach to improve mitochondrial respiration, especially for elderly patients.

With the development of autophagy research in the past decade, numerous autophagy modulators have emerged. Understanding the relationship between autophagy and age-related hepatic changes may lead to novel strategies to “rejuvenate” the aged liver. However, modulation of autophagy via pharmacological intervention is a promising but double-edged treatment strategy. Therefore, to effectively counteract liver aging without causing obvious harm, it is necessary to evaluate the most destructive process in the individual patient before modulating autophagy.

## Figures and Tables

**Table 1:** Induction of autophagy reduces hepatic steatosis.

Recent scientific evidence that activating autophagy improves liver steatosis							
Author year	Research model	Autophagy pathway	Autophagy modulation	Enhanced autophagy	Reduced steatosis	Reduced autophagy	Increased steatosis
Tong et al. [179] 2019	HFD-fed C57BL/6 mice; ob/ob mice; primary mouse hepatocytes; HepG2 cells	AMPK-mTOR	PPAR*δ* Chloroquine Atg5-KD	LC3-II: +++ P62: ---	TG: --- HE: ---	P62: +++	TG: +++ HE: +++
Ren et al. [176] 2019	HFD-fed C57BL/6 mice; ob/ob mice; Palmitate-stimulated HepG2 cells	AMPK-TFEB	Catalpol Chloroquine	LC3-II: +++ P62: ---	TG: --- TC: --- HE: --- Oil Red O: ---		TG: +++ TC: +++ Oil Red O: +++
Wang et al. [180] 2019	HFD and MCDD-fed C57BL/6J mice; Palmitate-stimulated primary mouse hepatocytes and HepG2 cells	AMPK-SIRT1	Tangshen formula SIRT1-KD	LC3-II: +++ P62: ---	TG: --- TC: --- HE: --- Oil Red O: ---	LC3-II: --- P62: +++	LDs: +++
Chu et al. [181] 2019	Oleic acid-stimulated HepG2 and LO2 cells	AMPK-mTOR Akt-mTOR	Cherry anthocyanins 3-Methyladenine; Atg5-KD	LC3-II: +++ P62: ---	TG: --- TC: --- Oil Red O: ---	LC3-II: ---	TG: +++ TC: +++ Oil Red O: +++
Ohashi et al. [182] 2019	HFD-fed male BALB/c mice	Not investigated	Conophylline	LC3-II: +++ P62: ---	TG: --- HE: --- Oil Red O: ---		
Liu et al. [183] 2018	HFD-fed male SD rats; Palmitate-stimulated L02 cells	COX-2	Celecoxib Rapamycin Chloroquine	LC3-II: +++ P62: ---	TG: --- Oil Red O: ---	LC3-II: --- P62: +++	TG: +++ Oil Red O: +++
Hong et al. [184] 2018	Male ob/ob and C57BL/6 mice; Palmitate-stimulated HepG2 cells and primary hepatocytes	SIRT1	Erythropoietin SIRT1-KD	LC3-II: +++	TG: --- Oil Red O: ---	LC3-II: ---	TG: +++ Oil Red O: +++
Balachander et al. [185] 2018	Oleic acid-stimulated HepG2 cells	Not investigated	Rosmarinic acid	LC3-II: +++	TG: --- TC: --- Oil Red O: ---		
Li et al. [186] 2017	HFD-fed male C57BL/6 mice; free fatty acid-stimulated HepG2 cells	Atg16L1-mediated	1,25(OH)_2_D_3_ 3-Methyladenine Atg16L1-KD	LC3-II: +++ P62: ---	TG: --- TC: --- HE: --- Oil Red O: ---	LC3-II: ---	TG: +++ TC: +++ Oil Red O: +++
Tang et al. [173] 2016	Chronic ethanol-fed male C57BL/6J mice; oleic acid and alcohol-stimulated HepG2 cells	Not investigated	Resveratrol 3-Methyladenine	LC3-II: +++ P62: ---	TG: --- HDL-C: +++ LDL-C: --- LDs: --- HE: --- Oil Red O: ---	LC3-II: --- P62: +++	TG: +++ Oil Red O: +++
Jung et al. [187] 2015	HFD-fed male C57BL/6J mice; Palmitate or tunicamycin-stimulated HepG2 cells and human primary hepatocytes	AMPK-mediated	C1q/TNF-related protein 9 Compound C 3-Methyladenine	LC3-II: +++ P62: ---	TG: --- HE: --- Oil Red O: ---	LC3-II: --- P62: +++	TG: +++ Oil Red O: +++
Zhang et al. [188] 2015	HFD-fed 129/SvJ mice; Palmitate-stimulated HepG2 cells	cAMP-PRKA-AMPK-SIRT1	Resveratrol 3-Methyladenine	LC3-II: +++ P62: ---	TG: --- Oil Red O: ---	LC3-II: --- P62: +++	TG: +++

+++: increase; ---: decrease; KO: knockout; KD: knockdown; HFD: high-fat diet; MCDD: methionine choline-deficient diet; TG: triglyceride; TC: total cholesterol; HDL-C: high-density lipoprotein cholesterol; LDL-C: low-density lipoprotein cholesterol; LDs: lipid droplets; PPAR*δ*: peroxisome proliferator-activated receptor *δ*; TFEB: transcription factor EB; cAMP: cyclic adenosine monophosphate; PRKA: protein kinase A; SIRT1: sirtuin 1; HE: hematoxylin and eosin stain; Oil Red O: Oil Red O stain.

**Table:** (a) Recent scientific evidence that activating autophagy aggravates liver fibrosis

Author year	Research model	Autophagy pathway	Autophagy modulation	Enhanced autophagy	Increased fibrosis	Reduced autophagy	Reduced fibrosis
Ma et al. [217] 2020	CCl_4_-stimulated male Norway rats; platelet-derived growth factor-BB (PDGF-BB) stimulated LX-2 cells	Not investigated	Small heterodimer partner			P62: +++ Atg12: ---	SMA: ---
Liu et al. [218] 2019	CCl_4_ and BDL-stimulated male C57 mice	TGF-*β*1-Smad3	Isorhamnetin CCl_4_ BDL	LC3-II: +++ Beclin-1: +++	*α*-SMA: +++ Hydroxyproline: +++ PPAR-*γ*: --- HE: +++ Masson: +++	LC3-II: --- Beclin-1: ---	*α*-SMA: --- Hydroxyproline: --- PPAR-*γ*: +++ HE: --- Masson:---
Meng et al. [219] 2018	LX-2 cells	Not investigated	Carvedilol Rapamycin	LC3-II/I: +++	Cleaved PARP: ---	Autophagic flux: ---	*α*-SMA: --- CCK-8: --- Bcl-2: --- Bax: +++ Cleaved PARP: +++
Feng et al. [220] 2018	CCl_4_ and BDL-stimulated male C57 mice	TGF*β*1-Smad3	Salidroside CCl_4_ BDL	LC3-II: +++ P62: --- Beclin-1: +++	*α*-SMA: +++ Hydroxyproline: +++ HE: +++ Masson:+++	LC3-II: --- P62: +++ Beclin-1: ---	*α*-SMA: --- Hydroxyproline: --- HE: --- Masson:---
Wang et al. [221] 2017	CCl_4_-stimulated female BALB/c mice; LX-2 cells	NF-*κ*B	3-Methyladenine Atg5-KD Rapamycin CCl_4_	LC3-II: +++ Beclin-1: +++	*α*-SMA: +++ TGF-*β*: +++ HE: +++ Masson: +++	LC3-II: --- Beclin-1: ---	*α*-SMA: --- TGF-*β*: --- HE: --- Masson: ---
Wu et al. [222] 2017	CCl_4_ and BDL-stimulated male C57 mice	TGF-*β*1-Smads PI3K-AKT	Quercetin CCl_4_ BDL	LC3-II: +++ P62: --- Beclin-1: +++	*α*-SMA: +++ Hydroxyproline: +++ HE: +++ Masson: +++	LC3-II: --- P62: +++ Beclin-1: ---	*α*-SMA: --- Hydroxyproline: --- HE: --- Masson: ---
Mao et al. [223] 2015	CCl_4_ and BDL-stimulated male C57BL/6 mice; HSC cell line	Not investigated	Ghrelin CCl_4_ BDL	LC3-II: +++ P62: ---	*α*-SMA: +++ Hydroxyproline: +++ HE: +++ Masson: +++	LC3-II: --- P62: +++	*α*-SMA: --- Hydroxyproline: --- HE: --- Masson: ---
Hernández-Gea et al. [201] 2012	CCl_4_ or TAA-stimulated C57BL/6 mice; mouse hepatic stellate cells; mouse stellate cell line JS1	Not investigated	3-Methyladenine Chloroquine Atg5/7-KD CCl_4_ TAA	LC3-II: +++ P62: ---	IPF: +++	LC3-II: --- P62: +++	*α*-SMA: --- Sirius Red: ---
Thoen et al. [198] 2011	Balb/c mouse; human and mouse HSCs	Not investigated	Bafilomycin A1 CCl_4_	Autophagic flux: +++	a-SMA:+++	Autophagic flux: ---	SMA: --- PDGFR-*β*: --- EdU: ---

**Table:** (b) Activating autophagy alleviates liver fibrosis

Author year	Research model	Autophagy pathway	Autophagy modulation	Enhanced autophagy	Reduced fibrosis	Reduced autophagy	Increased fibrosis
Liu et al. [224] 2018	CCl_4_-stimulated male SD rats; primary HSCs	Not investigated	Catalpol	LC3-II: +++ P62: --- Beclin-1: +++ Atg5: +++	*α*-SMA: --- Hydroxyproline: --- HE: --- Masson: --- Sirius Red: ---		
Ruart et al. [214] 2018	CCl_4_-stimulated C57BL/6 mice; LSECs	Not investigated	Atg7-KO			LC3-II/I: --- P62: +++	*α*-SMA: +++ Hydroxyproline: +++ Sirius Red: +++
Lodder et al. [213] 2015	CCl_4_-stimulated mice; Kupffer cells	Not investigated	Atg5-KO			LC3-II: --- P62: +++	*α*-SMA: +++ Sirius Red: +++

+++: increase; ---: decrease; KO: knockout; KD: knockdown; CCl_4_: carbon tetrachloride; TAA: thioacetamide; BDL: bile duct ligation; *α*-SMA: *α*-smooth muscle actin; PPAR-*γ*: peroxisome proliferator-activated receptor *γ*; PARP: poly(ADP-ribose) polymerase; HE: hematoxylin and eosin stain; Masson: Masson's trichrome stain; PDGFR-*β*: platelet-derived growth factor receptor type-b; IPF: idiopathic pulmonary fibrosis; EdU: 5-ethynyl-2′-deoxyuridine; LSECs: liver sinusoidal endothelial cells.

**Table:** (a) Activating autophagy via the mTOR-independent pathway facilitates liver regeneration

Author year	Research model	Autophagy pathway	Autophagy modulation	Enhanced autophagy	Increased regeneration	Reduced autophagy	Reduced regeneration
Guha et al. [266] 2019	Mice; MEFs; HEK293T cells	IPMK-AMPK-ULK1; IPMK-AMPK-SIRT1	IPMK-KO			LC3-II: ---	Ki-67: --- Edu: ---
Jia et al. [267] 2019	Male SD rats	Not investigated	70% PVL	LC3-II: +++	Cyclin D1: +++		
Liu et al. [10] 2018	Male SD rats, primary rat hepatocytes	Not investigated	Young plasma Wortmannin 3-Methyladenine	LC3-II: +++ p62: ---	Ki-67: +++	LC3-II: ---	Ki-67: ---
Wang et al. [268] 2017	Male C57BL/6 mice; AML12 cell line	Not investigated	TSG-6 3-Methyladenine	LC3-II: +++ Atg3: +++ Atg7: +++	Ki-67: +++ LBWR: +++	LC3-II: --- Atg3: --- Atg7: ---	CellTiter Proliferation Assay: --- LBWR: ---
Lin et al. [265] 2015	Male C57BL/6 mice	mTOR-independent	Amiodarone Atg7-KD	LC3-II: +++ p62: ---	Ki-67: +++ LBWR: +++	LC3-II: --- Atg7: ---	Ki-67: --- LBWR: ---
Cheng et al. [251] 2015	Liver progenitor cells	Not investigated	Beclin-1 overexpression Beclin-1-KD Atg5-KD	LC3-II: +++	PAS: +++	LC3-II: --- p62: +++ Beclin-1: --- Atg5: ---	PAS: --- CCK-8: ---
Toshima et al. [257] 2014	Mice	Not investigated	Atg5-KO			LC3-II: --- p62: +++ Atg5: ---	BrdU: ---

**Table:** (b) Activating autophagy via the mTOR-dependent pathway impairs liver regeneration

Author year	Research model	Autophagy pathway	Autophagy modulation	Enhanced autophagy	Reduced regeneration	Reduced autophagy	Increased regeneration
Shi et al. [269] 2018	Balb/c mice	mTOR-dependent	Rapamycin ASPP2-haploinsufficient	LC3-II: +++	PCNA: --- LBWR: ---	LC3-II: --- p62: +++	PCNA: +++ LBWR: +++
Fouraschen et al. [208] 2013	Male C57BL/6J mice	mTOR-dependent	Rapamycin & steroid dexamethasone	LC3-II: +++	PCNA: --- BrdU: --- LWRR: ---		
Kawaguchi et al. [260] 2013	Male C57BL/6J mice	mTOR-dependent	Temsirolimus		PCNA: --- LBWR: ---		
Espeillac et al. [102] 2011	Male C57BL/6J mice	mTOR-dependent	Temsirolimus		BrdU: ---		
Palmes et al. [258] 2008	Male Lewis rats	mTOR-dependent	Rapamycin		Ki-67: ---		
Jiang et al. [259] 2001	Male SD rats	mTOR-dependent	Rapamycin		LWRR: ---		

+++: increase; ---: decrease; KO: knockout; KD: knockdown; IPMK: inositol polyphosphate multikinase; SIRT1: sirtuin 1; ULK1: Unc-51 like autophagy activating kinase 1; PVL: portal vein ligation; LBWR: liver to body weight ratio; LWRR: liver-weight recovery rate; TSG-6: tumor necrosis factor-inducible gene 6 protein; PAS: periodic acid–Schiff stain.

**Table 4:** Induction of autophagy alleviates hepatic mitochondrial dysfunction.

Recent scientific evidence that activating autophagy improves liver mitochondrial dysfunction							
Author year	Research model	Autophagy pathway	Autophagy modulation	Enhanced autophagy	Improved mitochondrial function	Reduced autophagy	Increased mitochondrial dysfunction
Li et al. [307] 2020	HFD-fed male mice; palmitic acid-stimulated AML-12 cells; primary human hepatocytes	PINK1-Parkin	Cyanidin-3-O-glucoside	PINK1: +++ Parkin: +++ p62: ---	CPT1A: +++ SOD: +++ GSH-PX: +++ H_2_O_2_: --- MDA: --- IL-1B: ---		
Shan et al. [308] 2019	Acetaminophen-stimulated male C57/BL6 mice	PINK1-Parkin	Rapamycin Chloroquine	LC3-II/I: +++ p62: ---	MA: --- IL-1B: --- NLRP3: ---	p62: +++	IL-1B: +++ NLRP3: +++
Yu et al. [309] 2019	Palmitic acid and lipopolysaccharide-stimulated HepG2 cells	PINK1	Liraglutide 3-Methyladenine PINK1-KD	PINK1-FL: +++ Parkin: +++	ROS: --- IL-1B: --- NLRP3: --- ATP: +++	PINK1: ---	NLRP3: +++
Zhou et al. [310] 2019	HFD-fed male mice; palmitic acid-stimulated primary hepatocytes	AMPK-Parkin	Macrophage stimulating 1-KO	LC3-II/I: +++ Parkin: +++	∆*Ψ*m: +++ ROS: ---		
Liu et al. [311] 2018	HFD-fed male C57BL/J mice; oleate/palmitate-stimulated HepG2 cells	PINK1-Parkin	Quercetin	LC3-II: +++ Parkin: +++	CPT1: +++ RCR: +++ ∆*Ψ*m: +++ MA: ---		
Zhou et al. [300] 2018	HFD-fed C57BL/6J mice; palmitic acid-stimulated primary hepatocytes	Bnip3	Melatonin	LC3-II: +++ Atg5: +++ Beclin1: +++	ATP: +++ ∆*Ψ*m: +++ OCR: +++		
Yu et al. [299] 2016	Ethanol diet-fed male C57BL/6J mice	AMPK-ERK2	Quercetin	Parkin: +++ VDAC1: +++	∆*Ψ*m: +++ MA: ---		
Williams et al. [312] 2015	Ethanol administration C57BL/6J mice	Parkin	Parkin-KO			MPG: ---	RCR: --- COX: --- MA: ---

+++: increase; ---: decrease; KO: knockout; KD: knockdown; MA: morphological abnormalities; ERK2: extracellular signal-regulated kinase 2; VDAC1: voltage-dependent anion channel 1; CPT1/1A: carnitine palmitoyltransferase 1/1A; PINK1-FL: PINK1 precursor; NLRP3: nucleotide-binding oligomerization domain, leucine-rich repeat-containing receptor-containing pyrin domain 3; RCR: respiratory control ratio; COX: cytochrome c oxidase; MPG: mitophagosomes; Bnip3: Bcl-2/E1B-19KD-interacting protein 3; OCR: oxygen consumption rate; H_2_O_2_: hydrogen peroxide; SOD: superoxide dismutase; GSH-PX: glutathione peroxidase; MDA: malondialdehyde; GSSG: glutathione disulfide.
